# Dual roles of IL-22 at ischemia-reperfusion injury and acute rejection stages of rat allograft liver transplantation

**DOI:** 10.18632/oncotarget.23266

**Published:** 2017-12-15

**Authors:** Yi Zhang, Xiaofei Wang, Liwei Mao, Di Yang, Weiwu Gao, Zhiqiang Tian, Mengjie Zhang, Xia Yang, Kuansheng Ma, Yuzhang Wu, Bing Ni

**Affiliations:** ^1^ Institute of Immunology, PLA, Third Military Medical University, Chongqing 400038, PR China; ^2^ Department of Pathophysiology and High Altitude Pathology, Third Military Medical University, Chongqing 400038, PR China; ^3^ Laboratory Department, 150th Hospital of PLA, Luoyang 471031, PR China; ^4^ Department of Hepatobiliary Surgery, Southwest Hospital, Third Military Medical University, Chongqing 400038, PR China; ^5^ Department of Oncology, 309th Hospital of PLA, Beijing 100091, PR China

**Keywords:** liver transplantation, IL-22, Th17 cells, Treg cells, chemokine

## Abstract

Interleukin-22 (IL-22) is a recently identified regulator of inflammation, but little is known about its role in liver transplantation. Therefore, in this study, we explored the roles and the underlying mechanisms of IL-22 in acute allograft rejection by using a rat allogeneic liver transplantation model. Results showed that allograft liver transplantation led to damage of the parent liver and to significantly increased IL-22 expression in the allograft liver and plasma of the recipient rats compared with the rats who received isografts. Moreover, the significantly increased IL-22 expression was accompanied by markedly increased level of phospho-STAT3 in the allogeneic liver tissues after transplantation. Of note, neutralization of the IL-22 protein in recipient rats significantly worsened the function of the allograft liver at 1 day post-transplantation (ischemia-reperfusion injury, IRI) but improved the function at 7 days post-transplantation (acute rejection, AR). At IRI stage, IL-22 protected liver function through the increase of anti-apoptosis and pro-regeneration cytokines. However, IL-22 led to the increase of pro-inflammation factors at AR stage, accompanied by the marked increase of the Th17 and the marked decrease of Treg cells in allograft recipient rats through modulating the expression of chemokines for different cell types, which however were reversed by *in vivo* IL-22 neutralization. Results indicate the dual roles of IL-22 and suggest the differential potential clinical application of IL-22 at different stage of allograft liver transplantation.

## INTRODUCTION

Liver transplantation is a widely accepted treatment for many end-stage liver diseases. [1] Although advancements in the surgical techniques and immunosuppressive agents have improved the outcomes of liver transplantation, acute rejection (AR) remains to be an important cause of dysfunction of the graft after transplantation. It is well known that T and B cells play important roles in the regulation of allograft rejection, and nonspecific immune cells, such as the natural killer (NK) cells, innate lymphoid cells (ILCs), macrophages and polymorphonuclear cells, have also been implicated as crucial contributors to the transplant rejection response. [2] One of the major mechanisms of transplant rejection involving these immune cells is the secretion of the type I and type 17 effector cytokines, such as interferon-gamma (IFNγ) and interleukin (IL)-17. [3–7] However, the role of another type 17 cytokine, IL-22, remains unclear in liver transplantation.

IL-22 is a recently characterized cytokine of the IL-10 superfamily, which is produced by αβ T cells, γδ T cells, natural killer T (NKT) cells and ILCs, [8, 9] and its function has been identified in numerous tissues. [10] Studies in experimental models of hepatitis, rheumatoid arthritis, colitis and thymic injury have demonstrated the protective function of IL-22 exerted by its promotion of epithelial tissue proliferation and regeneration. [10–13] However, IL-22 has also been demonstrated to be a contributor to inflammatory tissue pathology, through its induction of the expressions of multiple proinflammatory factors. [13–15] In transplantation, IL-22 has also been reported to play inconsequent roles. Hanash et al. [16] reported that IL-22 plays protective roles by decreasing tissue damage and mortality in graft versus host disease (GVHD); however, others have reported that IL-22 leads to acute GVHD by aggregating effector T cells and reducing the regulatory T cells (Treg), and that IL-22 deficiency attenuates murine acute GVHD mortality. [17–19] Other recent studies have indicated that IL-22 plays protective roles in liver regeneration and liver ischemia-reperfusion injury (IRI). [20, 21] Nevertheless, whether and how IL-22 functions in AR of liver transplantation is unknown as of yet.

In this study of a rat allogeneic liver transplantation model, we found that IL-22 expression was significantly up-regulated, which exerted a protective role at IRI stage (at 1 day post transplantation) but played a pathogenic role at AR stage (at days 5-7 post transplantation) of the allograft liver transplantation through alteration of the Th17/Treg balance in recipient rats, likely involving activation of the signaling pathway mediated by the signal transducer and activator of transcription factor 3 (STAT3).

## RESULTS

### Liver allograft induced a significant acute rejection response in rats

**Figure 1:**
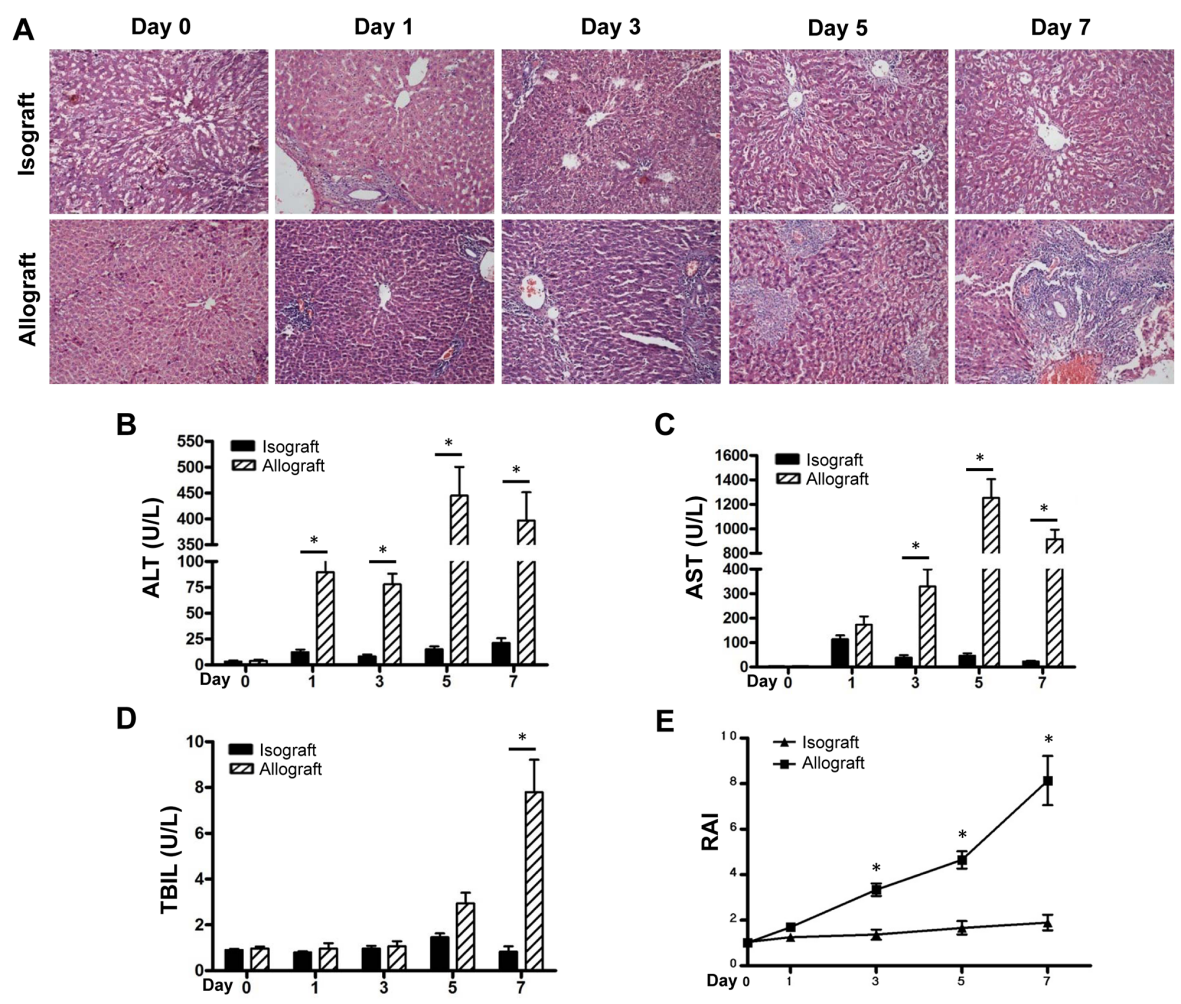
AR responses in the isograft and allograft groups Recipient rats in the isograft group (BN-to-LEW) and the allograft group (LEW-to-BN) were sacrificed before or after liver transplantation at days1, 3, 5 and 7 (5 rats at each time point for each group). Morphology of the transplanted mouse liver was observed by HE staining **(A)**. Plasma ALT levels **(B)**, AST levels **(C)** and TBIL levels **(D)** were detected at the indicated times. Histological classification was analyzed by RAI according to Banff’s scheme **(E)**. ^*^p<0.05.

Compared to the recipients of the BN-to-LEW isograft, recipients of the LEW-to-BN allograft [22] presented with inactivity, weakness and anorexic. HE staining showed a serious and progressive AR response in liver tissues of the allograft group, which was characterized by severe hepatocyte necrosis, leucocyte infiltration and bile duct damage and peaked during the 5-7 days post transplantation; few histological changes were shown in the isograft group (Figure 1A). The similar results were observed for the impaired liver function during AR stage, including the plasma levels of ALT and AST compared with the isograft group (Figure 1B and 1C). The TBIL level also showed an increase postoperatively, with a particularly sharp increase at day 7 (Figure 1D). RAI scores in the allograft group after 3 days postoperatively increased significantly, as compared to those in the isograft group (Figure 1E).

### IL-22 expression is markedly up-regulated at AR stage in allogeneic liver transplantation

**Figure 2:**
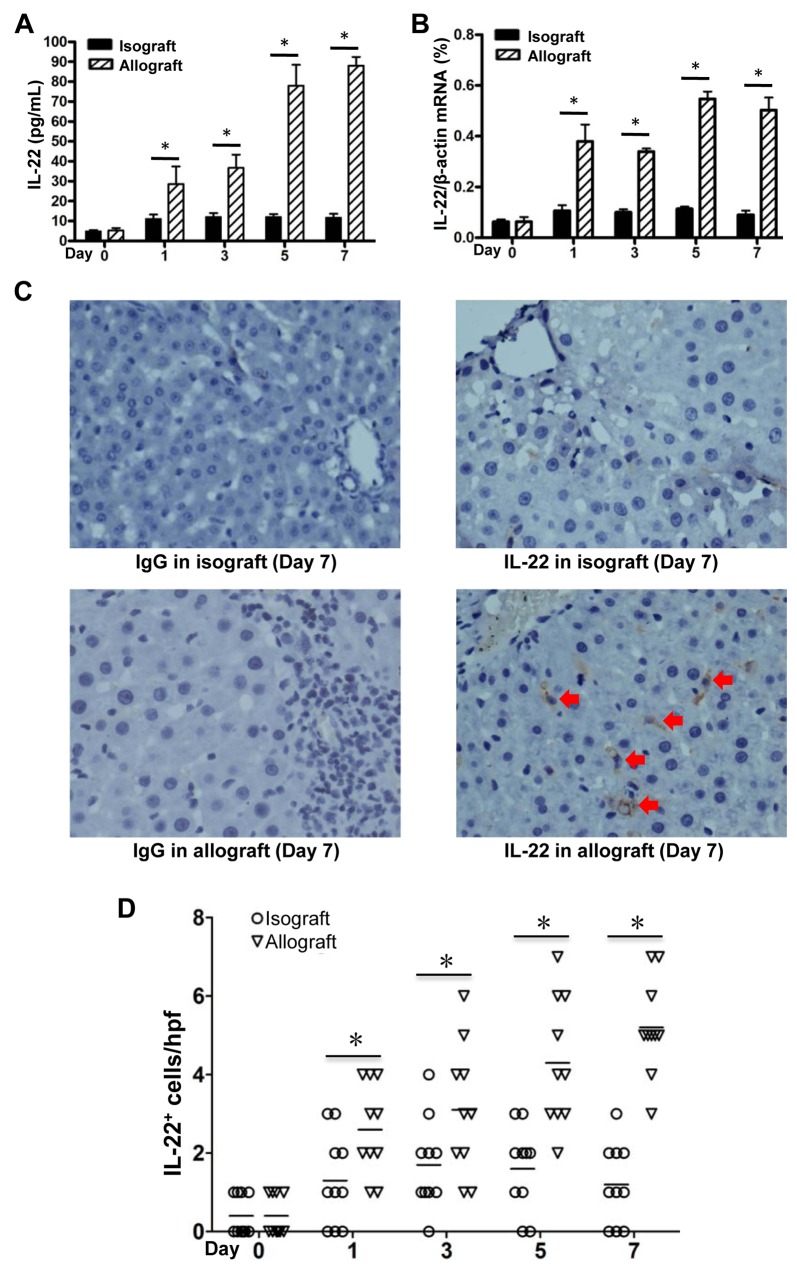
IL-22 expression in allogeneic and isogenic liver transplant tissues IL-22 concentrations in plasma **(A)** and IL-22 mRNA levels in liver tissues **(B)** of recipients (n=5) in the isograft group and the allograft group before or at the 1, 3, 5 and 7 days after liver transplantation. **(C)** IHC assay for IL-22 expression in liver tissues of recipients in the allograft and isograft groups at 7 days postoperatively, with isotype IgG as control staining. Positive cells were indicated by red arrows. **(D)** Statistical analysis of liver portal areas IL-22^+^ cell counts in IHC staining tissues. Five different high power fields (hpf) (400 ×) of liver portal areas were used for counting positive cells by two independent observers. ^*^ *p*<0.05.

IL-22 has been proven to play protective roles in liver regeneration and liver IRI. [20, 21] In order to assess the contribution of IL-22 in the process of liver transplantation, we detected the IL-22 concentrations in plasma and IL-22 mRNA levels in liver tissues of recipient rats. We found a gradually but significantly increased IL-22 expression in the allograft group from 1 day to 7 days, and peaked during 5-7 days post transplantation, as compared with the day 0 group and the time point-paired isograft group (Figure 2A and 2B). IHC further demonstrated there were much more IL-22 positive cells infiltrated in the allograft liver tissues compared with those in the isografted liver tissues at 7 days (Figure 2C and 2D).

### IL-22 was mainly derived from the CD4^+^T cells in allograft liver transplantation

**Figure 3:**
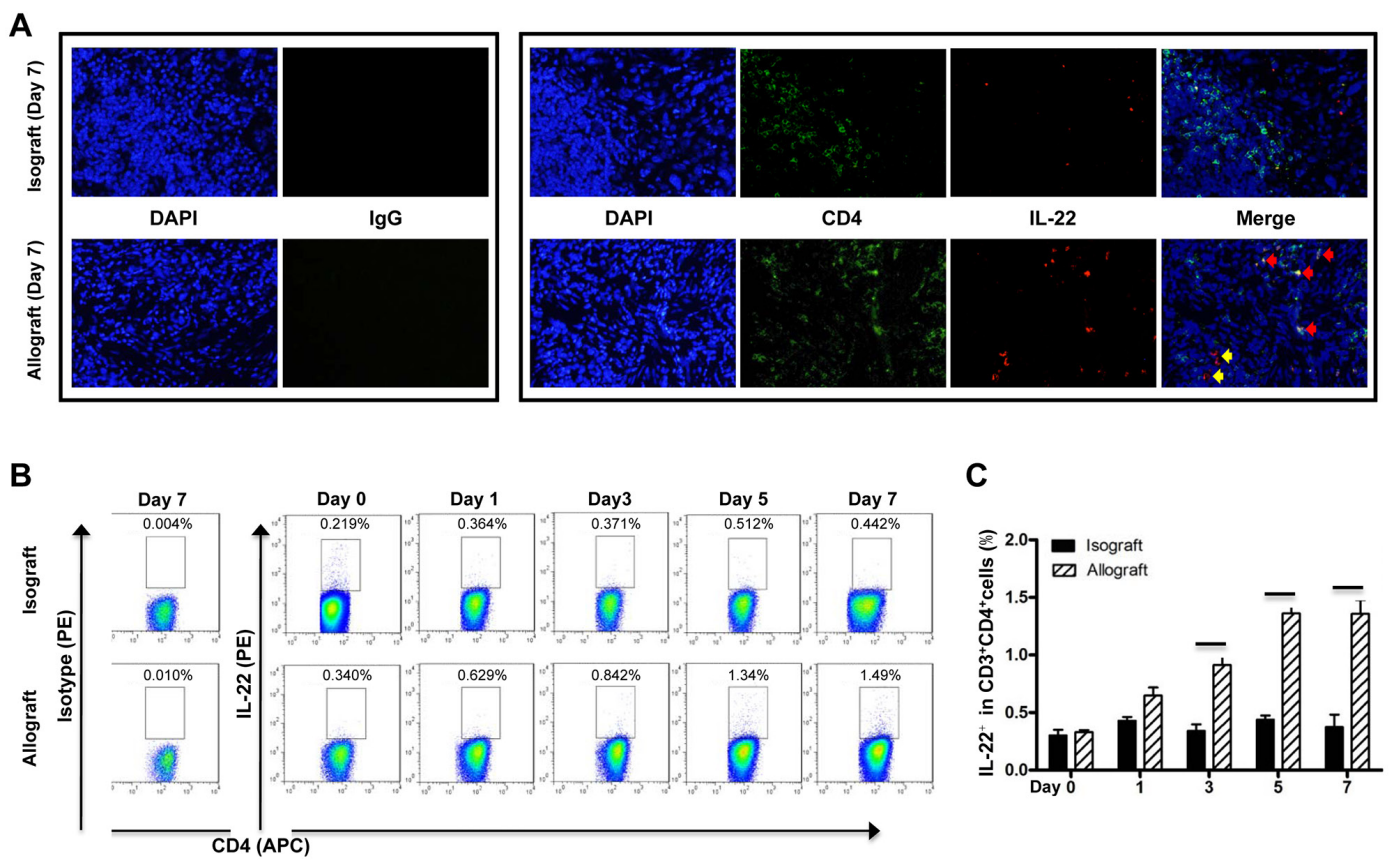
Cellular source of IL-22 in liver transplantation **(A)** Representative IF staining of IL-22 and CD4 in liver tissues of the allograft group at 7 days postoperatively (n=5). The representative CD4 and IL-22 double positive cells and IL-22 positive cells were indicated by red arrows and yellow arrows, respectively. **(B and C)** Flow cytometry assay of IL-22 expression. Liver lymphocyte from rats of the isograft group (n=5) and allograft group (n=5) at the indicated times after liver transplantation were *in vitro* stimulated with PMA and ionomycin. After CD3 gating, the frequency of CD4 and IL-22 positive cells was analyzed by flow cytometry. The representative flow cytometry results (B) and the statistical results (C) are shown. ^*^p<0.05.

IL-22 can be produced by several immune cells, mainly CD4^+^T cells and ILCs. [8, 9] In the allografted livers of the recipients at 7 days post transplantation, most IL-22 protein was expressed in the CD4 positive cells, as shown by multiple IF staining assays (Figure 3A), suggesting CD4^+^T cells might be the major source of IL-22 protein in allografted liver tissue. In contrast, there were only minimal IL-22^+^ or IL-22^+^CD4^+^ cells detected in the isografted livers (Figure 3A). Flow cytometry further revealed that the frequency of IL-22^+^CD4^+^T cells was significantly increased in the allograft group from 3 days to 7 days after liver transplantation, while no significant changes in the frequency of IL-22^+^CD4^+^T cells were observed in the isograft group compared to the control rats without operation (Figure 3B and 3C).

### IL-22 plays a protective role at IRI stages versus a pathogenic role at AR stage of allograft liver transplantation

**Figure 4:**
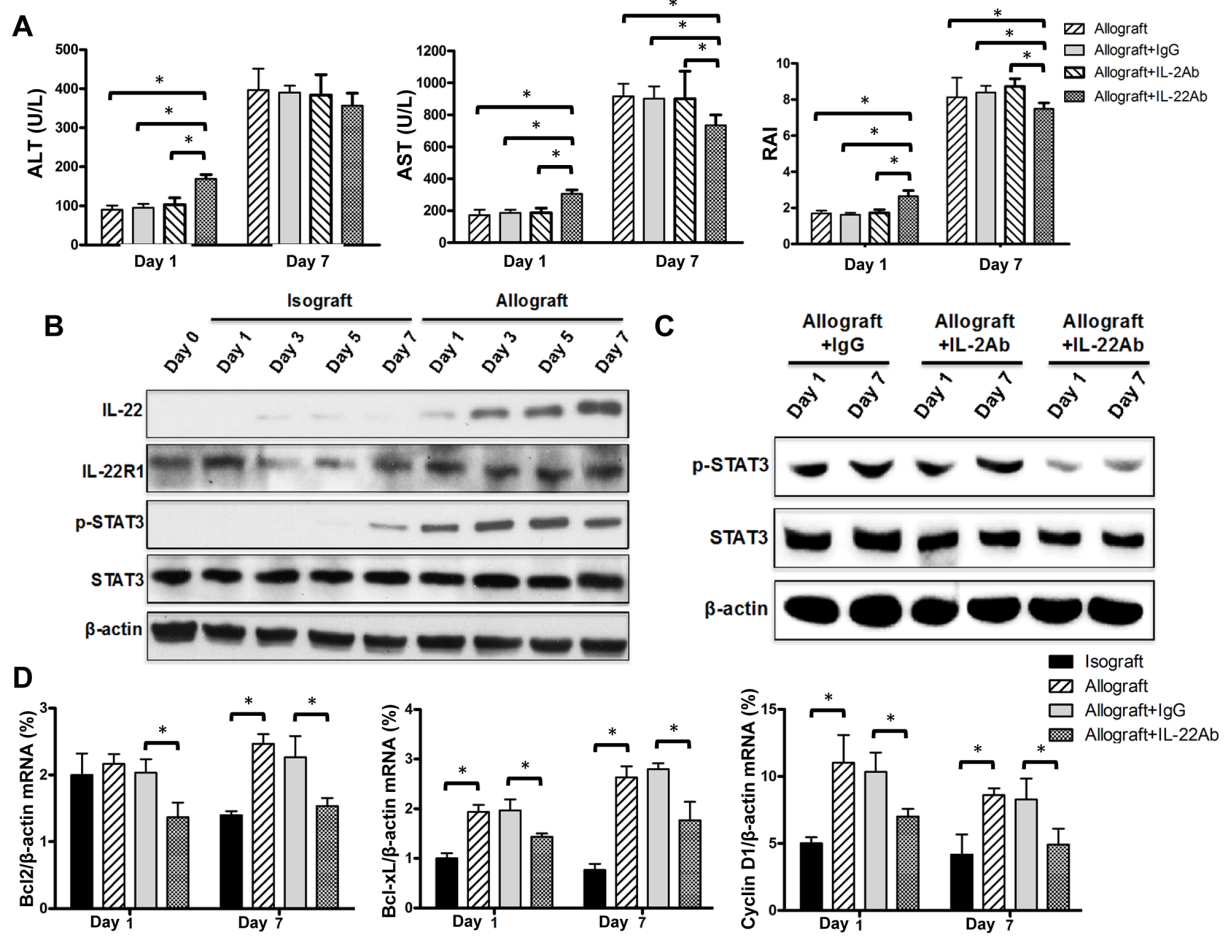
Reverse effects of IL-22 at IRI and AR stages of allograft liver transplantation **(A)** Recipient rats (n=5) of the allograft group were treated with the indicated neutralizing antibodies by intravenous injection twice at 12 and 24 h prior to sacrifice at 1 day and 7 day post-transplantation, respectively. Plasma ALT levels, AST levels, and RAI scores were determined for the indicated groups at the indicated times. **(B and C)** Total protein was extracted from liver tissue and the expressions of IL-22, IL-22 R1, p-STAT3 and total STAT3 proteins at the indicated groups and time points were evaluated by Western blotting with β-actin as the internal control. **(D)** Bcl2, Bcl-xL and Cyclin D1 mRNA levels in liver tissues of recipients (n=5) in the indicated groups at days 1 and 7 after liver transplantation were evaluated by quantitative PCR. ^*^p<0.05.

The markedly increased IL-22 expression observed in recipients after allograft liver transplantation might exert protective or pathogenic effects on transplanted livers. To clarify this issue, we sought to further define the exact role of the significantly increased IL-22 expression in allogenic liver transplantation by performing pretreatment of recipient rats with the neutralizing IL-22 antibody at 12 h and 24 h before sacrifice at days 1 and 7 post-transplantation, respectively. Results showed that rats treated with the neutralizing IL-22 antibody at 1 day post-transplantation demonstrated significantly elevated levels of ALT and AST, and significantly higher RAI score compared with the control rats pretreated with isotype IgG (Figure 4A), suggesting a protective role of IL-22 at IRI stage. Regardless, the isograft liver transplantation showed no difference for these indexes with the IgG control group (date not shown). However, IL-22 blockade significantly decreased the AST level and RAI score at 7 days post-transplantation compared with the isotype control (Figure 4A), suggesting a detrimental role of IL-22 at AR stage.

It has been reported that IL-22 induces STAT3 activation in the liver and IL-22 blockade significantly reduces hepatic STAT3 activation in T cell-mediated hepatitis. [23] In this study, we found that the gradually but markedly increased IL-22 level post-transplantation was accompanied by remarkably increased phosphorylated (p)-STAT3 level in allograft liver tissues (Figure 4B). However, only minimal IL-22 and p-STAT3 were detected in isograft liver tissues compared with the control rats without operation (Figure 4B). Furthermore, blockade of IL-22 significantly decreased the p-STAT3 level in liver tissue at day 1 and day 7 post-transplantation (Figure 4C). Results also showed that the expressions of IL-22R1 and IL-10R2 (subunits of the heterodimer IL-22 receptor), and the total STAT3 protein levels did not significantly alter at different time points in each group (Figure 4B-4C and Supplementary Figure 1).

Because the major effect of activation of STAT3 by IL-22 in liver tissue appears to be prevention of cellular apoptosis and promotion of cell survival and proliferation [24], we thus further detected the expression levels for apoptosis and proliferation related genes such as Bcl-2, Bcl-XL and CyclinD1in liver tissues of recipient rats at days 1 and 7 post transplantation. Compared with the isografted livers, the mRNA expressions of Bcl-2, Bcl-XL and Cyclin D1 were almost all markedly increased in allografted livers at day 1 post transplantation, however, IL-22 blockade markedly down-regulated their expression (Figure 4D). Interestingly, the similar results were observed at day 7 post transplantation (Figure 4D). These data indicated that IL-22 plays a protective role at IRI stage of liver transplantation through enhancing anti-apoptosis and pro-regeneration via the activation of STAT3, however, the detrimental effects of IL-22 on allografted liver during AR stage might attribute to other mechanisms rather than affecting apoptosis or proliferation of liver cells.

In addition, it has been well-known that IL-2 can also activate STAT3 pathway during T cell activation [25, 26], we then treated the experimental mice with neutralizing IL-2 antibody, which however did not cause significant alteration for the IRI and AR responses and the levels of p-STAT3, Bcl-2, Bcl-XL and CyclinD1 in liver tissues of recipient rats at days 1 and 7 post allograft liver transplantation (Figure 4). Furthermore, it is reported that CD4^+^T cells also secrete IL-13 to activate STAT3 pathway [27]. However, we found that IL-13 expression was increased only at 7 days post transplantation in the allograft group (Supplementary Figure 2), suggesting that IL-13 might involve in liver transplantation beyond AR stage.

### The detrimental effects of IL-22 at AR stage may be mediated through recruiting Th17 cells and decreasing Treg cells in allograft liver transplantation

**Figure 5:**
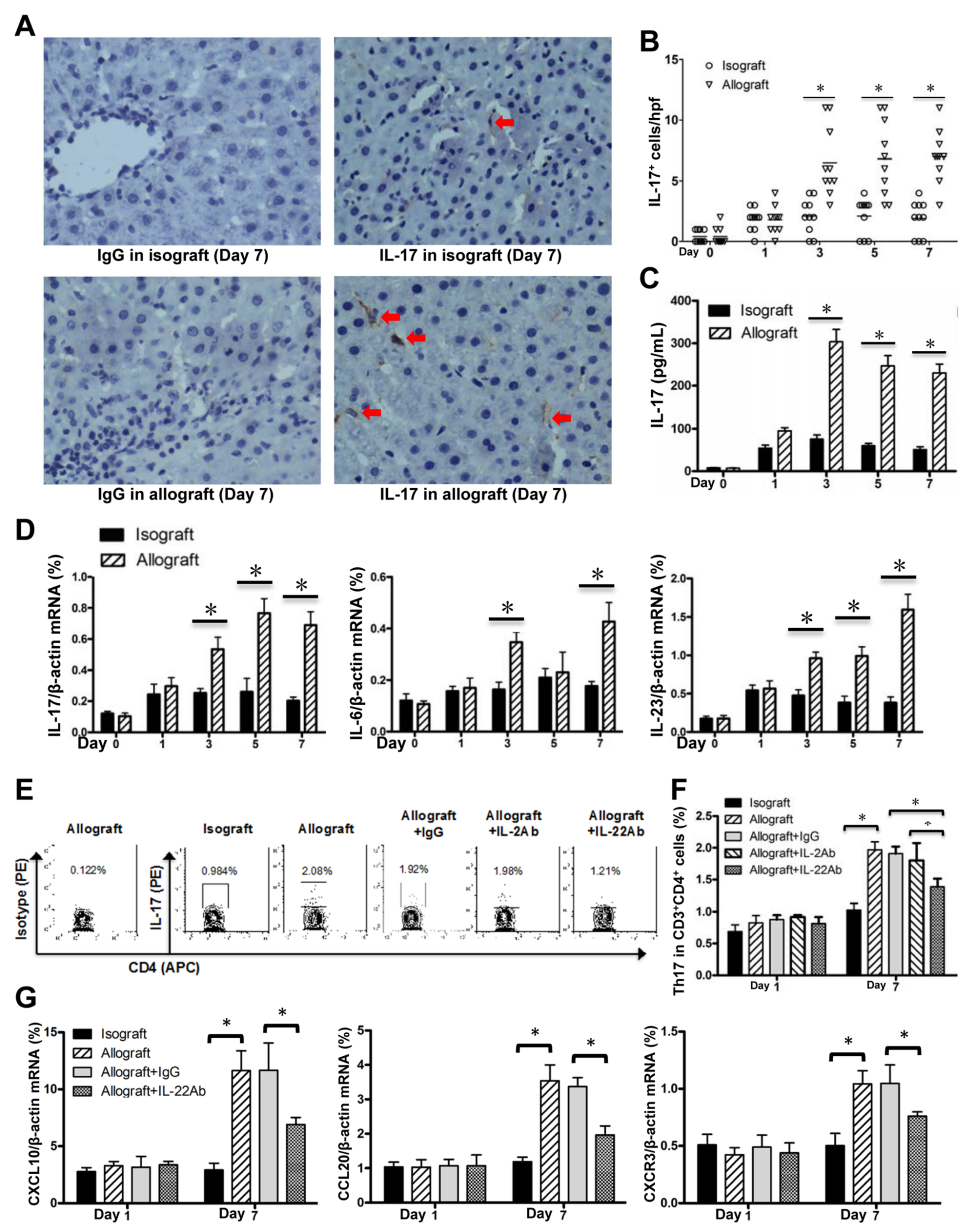
Enhanced Th17 cells in allografted liver tissues **(A)** IHC for IL-17 expression in liver tissues of recipients in the allograft and isograft groups at 7 days postoperatively. Positive cells were indicated by red arrows. **(B)** Statistical analysis of liver portal areas IL-17^+^ cell counts in IHC staining tissues. Plasma IL-17 concentrations **(C)** and mRNA levels of IL-17, IL-6 and IL-23 in liver tissues **(D)** of recipients (n=5) in the isograft or allograft group at the indicated time points were determined by ELISA or quantitative PCR. **(E)** The representative FCM results for the frequencies of Th17 cells (CD3^+^CD4^+^ IL-17^+^) in liver lymphocytes of the indicated groups at the 7 days after liver transplantation (n=5). **(F)** The statistical analysis for Th17 frequency in various groups at the indicated time points. **(G)** CXCL10, CCL20 and CXCR3 mRNA levels in liver tissues of recipients (n=5) in the indicated groups at days 1 and 7 after liver transplantation. ^*^p<0.05.

It has been known that Th17 cells and IL-17 plays a detrimental effect on liver transplantation [5]. In our study, IHC assay showed that IL-17 positive cells infiltrated the liver tissues of the allograft group at AR stage postoperatively, compared with the isograft group (Figure 5A and 5B). The levels of plasms IL-17 protein and liver IL-17 mRNA were significantly increased in the allograft group compared with the isograft group after 3 days post transplantation (Figure 5C and 5D). We also observed the Th17 differentiation related cytokines IL-6 and IL-23, were also increased in allograft liver transplantation (Figure 5D). FCM assay further confirmed the Th17 frequency was markedly increased at day 7 compared with day 1 post allograft liver transplantation (Figure 5E and 5F). To determine whether the detrimental effect of IL-22 is mediated through affecting Th17 frequency in allografted liver, we blocked the IL-22 by treatment of mice with the neutralizing IL-22 antibody. While the Th17 cell frequency was not changed by IL-22 blockade at day 1, we observed a markedly decreased Th17 cell frequency at day 7 after IL-22 blockade. IL-2 blockade did not alter Th17 cell frequency at 1 and 7 days post allograft liver transplantation (Figure 5E and 5F). In addition, it is reported that IL-22 stimulates the secretions of C-X-C motif ligand 10 (CXCL10), C-C motif ligand 20 (CCL20) and the C-X-C chemokine receptor 3 (CXCR3) to promote intrahepatic Th17 recruitment [28]. In this study, we observed that, compared with the isografted livers, the mRNA expressions of CXCL10, CCL20 and CXCR3 were increased in allografted livers at day 7 post transplantation, which however were markedly down-regulated with IL-22 blockade (Figure 5G).

**Figure 6:**
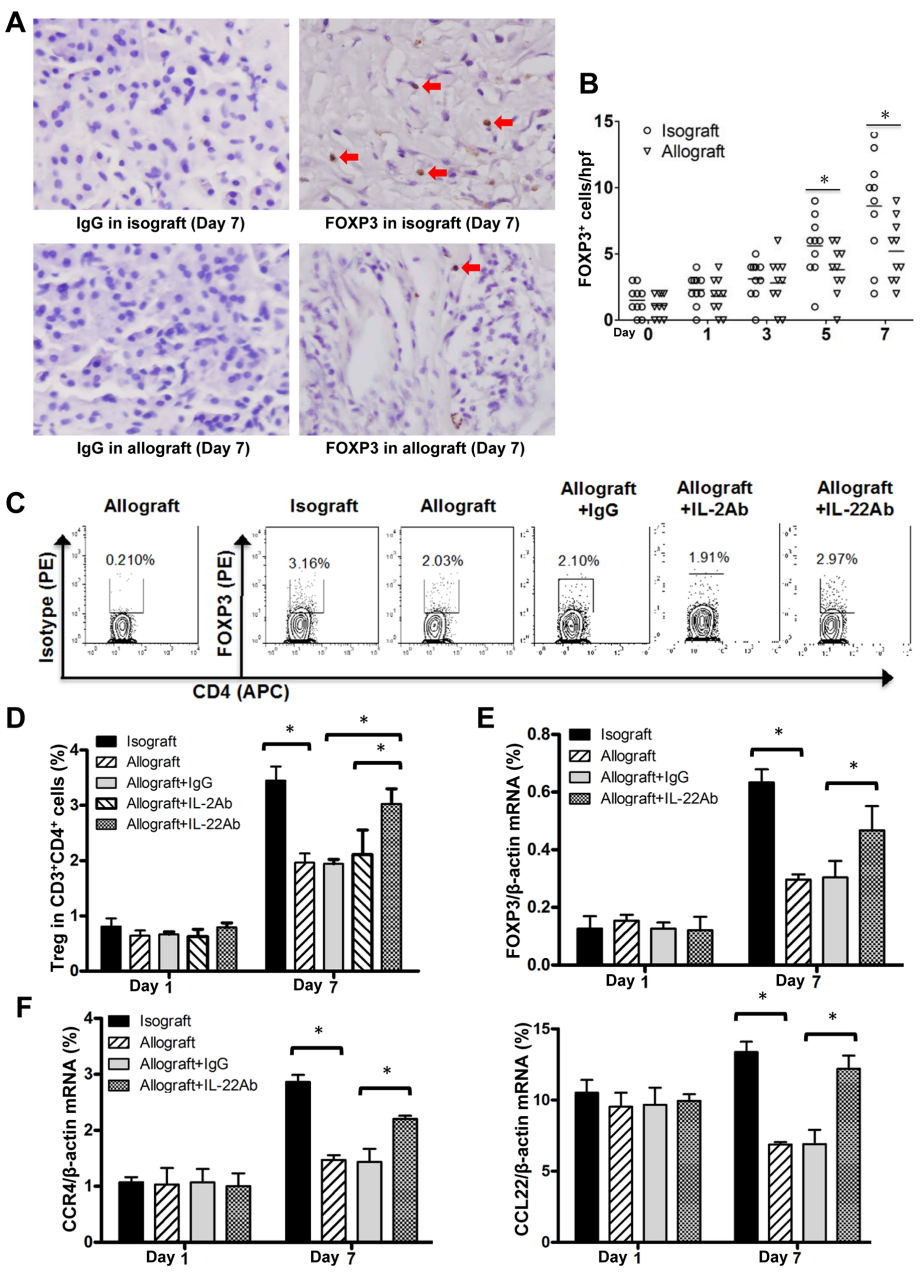
Decreased Treg cells at AR stage of allograft liver transplantation **(A)** IHC assay for Foxp3 positive cells in isografted or allografted liver tissues at 7 days postoperatively. Positive cells were indicated by red arrows. **(B)** Statistical analysis of liver portal areas Foxp3^+^ cell counts in IHC staining tissues. **(C)** The representative results of FCM assay for the frequency of Treg cells (CD3^+^CD4^+^Foxp3^+^) in liver lymphocyte of the indicated groups at the 7 day post transplantation. **(D)** The statistical analysis of Treg cell frequency in various groups at the indicated time points after liver transplantation (n=5). **(E and F)** Foxp3, CCR4 and CCL22 mRNA levels in liver tissues of recipients (n=5) in indicated groups at the indicated time points after liver transplantation. ^*^p<0.05.

In addition, it is reported that Treg cells have been implicated substantially in solid organ transplantation [29], we thus further explored whether Treg cells contribute to the detrimental effects of IL-22 on the acute rejection of allografted liver tissues. IHC and FCM assays demonstrated the markedly increased Foxp3^+^ or Treg cells in isografted liver tissues at 5 and 7 days post transplantation. In contrast, only relatively mild increase of Foxp3^+^ or Treg cells in allografted liver tissues were observed at these time points (Figure 6A-6D), which however were sharply increased after IL-22 blockade (Figure 6C and 6D). The mRNA expression of Foxp3 and the CCL22/CCR4 axis for the recruitment of Treg cells in allografted liver tissues was in accordance with the alteration trends of Treg cells at AR stage (Figure 6E-6F).

## DISCUSSION

Liver transplantation is an effective treatment option for end-stage liver diseases. However, acute rejection is still a crucial cause of graft loss and significantly decreases recipient post-transplant survival. [30] Several immune cells and cytokines contribute to this process. However, whether and how IL-22 is involved in liver transplantation has been unclear. In this study, by using the rat model of isograft and allograft liver transplantation, we determined that IL-22 is involved in the development of acute liver rejection, possibly via activation of the STAT3 pathway, and subsequent amelioration of the aberrantly altered Th17 and Treg balance after transplantation.

IL-22 is one of the key members of the IL-10 family of cytokines. Unlike most of the other IL-10 family members, IL-22 principally impacts the non-hematopoietic epithelial cells and fibroblasts in such diverse tissues as the gut, liver, lung, skin, thymus and kidney, due to IL-22R being expressed on epithelial cells and some fibroblasts present in those tissues and not on lymphoid cells [13]. In liver, the main target of IL-22 is hepatocytes. IL-22 can induce the hepatic production of acute-phase proteins, [31, 32] and induce proteins involved with anti-apoptosis and regeneration from damage. [23, 33, 34] Thus, IL-22 has been assigned a protective role in acute hepatic injuries such as T cell-mediated hepatitis, acute hepatitis, liver IRI, bacterial and parasitic infection. [20, 21, 33, 34] The beneficial effects of IL-22 on acute liver diseases were supported by our allograft liver transplantation study, which demonstrated the protective roles of IL-22 at 1 day post transplantation (IRI stage).

However, Jiang et al. [35] found that excessive expression of IL-22 during chronic hepatitis and hepatocellular carcinomas (HCCs) may allow survival of damaged hepatocytes that are precursors for HCCs, thereby promoting cancer. These results support the harmful effects of long-term and sustained IL-22 activation in a tumor and chronic hepatitis microenvironment. The current research indicated that persistent over-expression of IL-22 in the liver would be proinflammatory and pathogenic. Rats treated with neutralizing IL-22 antibody at IRI stage post-transplantation demonstrated significantly elevated ALT levels, AST levels and the RAI scores, which contrasted the results from rats treated with neutralizing IL-22 antibody at7 days postoperatively (AR stage) which showed significantly decreased AST level; thus, IL-22 might play a protective role at the IRI stage by increasing the regeneration and anti-apoptosis that would be beneficial towards relieving transplantation-associated liver IRI. However, this process might play a pathogenic role at the AR stage of allograft liver transplantation by producing some proinflammatory factors, such as Th17 related cytokines, which were found to be significantly increased at 7 days post-transplantation in the liver of the recipient rats in this study.

Interestingly, we observed the mechanisms underlying the beneficial effects of IL-22, which is probably mediated by prosurvival effects as observed at IRI stage, also existed at AR stage because we observed the enhanced expression of Bcl2, Bcl-xL and cyclin D1 in allografted liver tissues at AR stage compared with IRI stage. In addition, it is reported that STAT3 signaling activation is the key pathway for the functions of IL-22. [28, 36] Persistent activation of STAT3 can lead to up-regulation of proliferation-associated Cyclin D1, cell survival-associated Bcl-XL and Bcl-2, and VEGF-associated metastasis, thereby increasing tumor cell proliferation, survival and invasion [37]. In this study, we also observed the sustained increase of pSTAT3 level post allograft liver transplantation. However, IL-22 blockade markedly decreased the expression of these molecules, suggesting the detrimental effects of IL-22 at AR stage might be due to other mechanisms other than prosurvival effects. In addition, we observed in this study that the increased abundance of IL-22 at day 7 post-transplantation did not further augment levels of p-STAT3, suggesting that the pathogenic effect of IL-22 on allograft liver at AR stage could be partially independent of STAT3 activation. It is possible that IL-22 might also activate other STATs because it is reported that IL-22 also activates STAT1 and STAT5(https://cgap.nci.nih.gov/Pathways/BioCarta/h_il22bppathway), which have to be investigated in the future.

It is reported that Th17 cells play a pathological role and Treg cells play a detrimental role in liver transplantation, and thus Th17/Treg balance plays key roles in transplant rejection or tolerance [4]. This balance might be disturbed at AR stage of allograft liver transplantation. The results from this study confirmed this hypothesis because the frequency of Th17 cells was significantly higher than that in the isograft group, while the frequency of Treg cells in the allograft group was markedly lower than that in the isograft group at day 7 post-transplantation; however, there were no differences found between the isograft and allograft groups at day 1 post-transplantation. Such an imbalance in Th17/Treg cells might have been caused by the significantly increased IL-22 level at day 7 post-transplantation. In the current study, we found that neutralization of IL-22 caused a significant decrease in Th17 cells and a significant increase in Treg cells at 7 days post-transplantation, suggesting that IL-22 may alter the Th17/Treg balance by increasing Th17 cells and decreasing Treg cells to promote the acute transplant rejection at the AR stage of allograft liver transplantation.

It is reported that in clinics and mouse model of chronic hepatitis and fibrosis, blockade of IL-22 attenuated hepatic expression of CXC10 and CCL20 and subsequently reduced Th17 recruitment and liver inflammation and fibrosis progression. Blocking CXCR3 or CCL20 reduced Th17 cell chemotaxis by IL-22-treated HSCs [28]. In our study, we also observed the markedly increase of CXC10, CCL20 and CXCR3 in allografted liver tissues at 7 days post transplantation, furthermore, blockade of IL-22 significantly decreased the expression of these chemokines and the frequency of Th17 in allografted liver tissue. In addition, it is well known that CCR4 is predominantly expressed by effector Treg cells, and Treg migration and infiltration into various tumor tissues are dependent on the expression of CCR4 ligands including CCL22 produced by tumor cells or infiltrating macrophages [38]. It is also reported that IL-22 aggravates murine acute graft-versus-host disease by expanding effector T cells and reducing Treg cells though the underlying mechanisms remain unclear [17]. In our study, compared with the isografted livers, the mRNA expressions of CCR4 and CCL22 were much lower in allografted livers at day 7 post transplantation, which however were markedly up-regulated with IL-22 blockade. Therefore, the data from current study suggest that the markedly enhanced Th17 cells and decreased Treg cells in allografted liver tissues might be due to the increased chemokines for Th17 cell recruitment and the decreased chemokines for Treg cell recruitment by the sustained IL-22 induction, respectively, which finally contribute to the imbalance of Th17/Treg cells and the consequent detrimental effects of IL-22 on allograft liver transplantation at AR stage.

IL-22 can be derived from several cell types basing on the specific context of experimental models [9, 39]. Our multiple IF staining showed that most IL-22 protein was expressed in the CD4 positive cells in the allografted livers of the recipients at AR stage of liver transplantation. Furthermore, FCM assays demonstrated that the frequency of IL-22^+^CD4^+^T cells was significantly increased in the allograft group at AR stage, and the alteration of IL-22^+^CD4^+^T cells frequency was highly concordant with the alteration trend of IL-22 concentration during acute rejection after allograft liver transplantation, suggesting that IL-22 might be derived mainly from of CD4^+^T cells in the context of allograft liver transplantation. However, we also noticed that a few IL-22 positive cells could not merged with CD4 staining, indicating the multiple cellular source of IL-22 during allograft liver transplantation.

**Figure 7:**
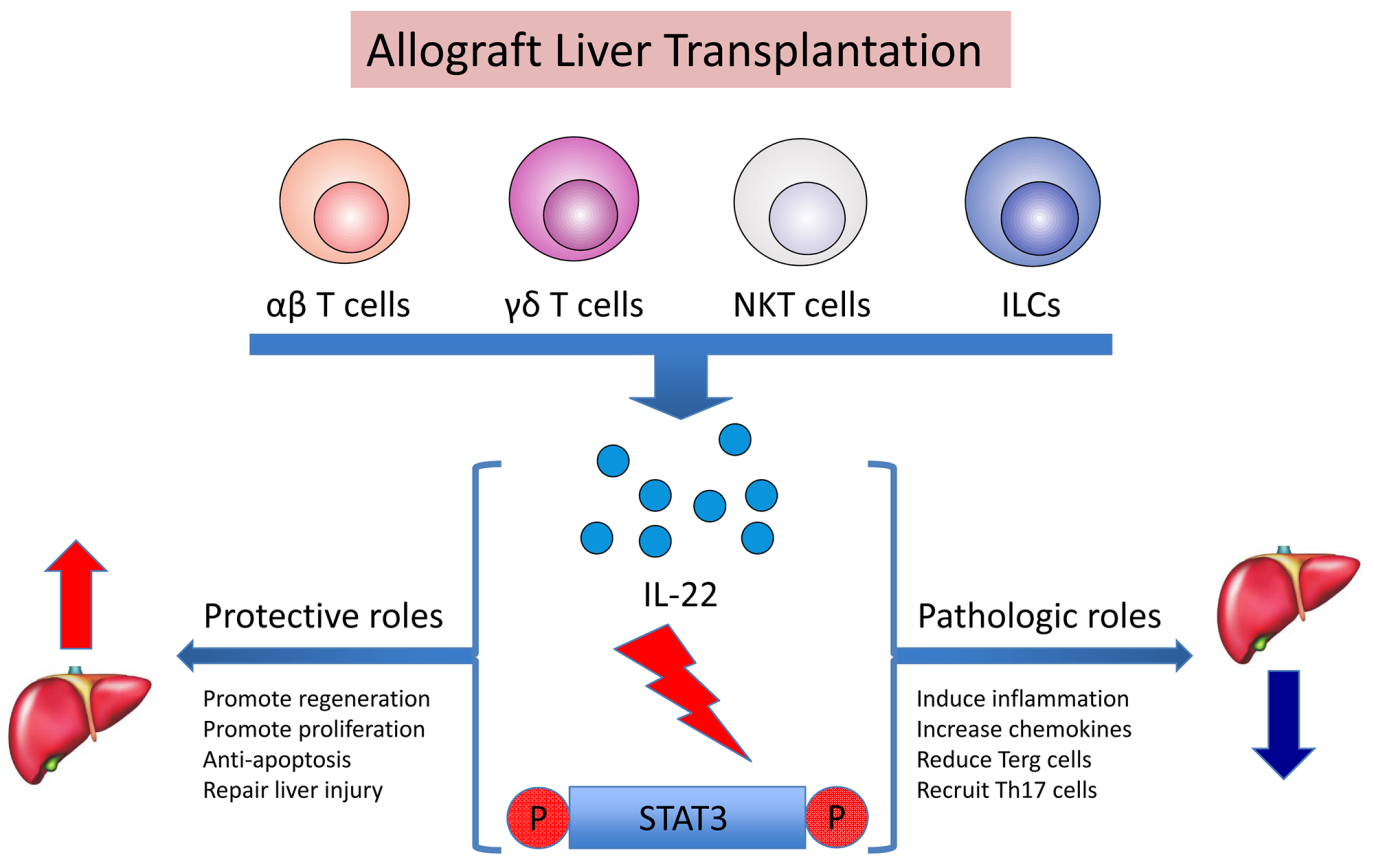
Dual roles of IL-22 in rat liver transplantation by the activation of STAT3 After allograft liver transplantation, IL-22 was secreted by multiple cell sources (and possibly αβ T cells, γδ T cells, NKT cells and ILCs) stimulated by the acute injection process. Through activation of the STAT3 signaling pathway, IL-22 plays a dual role towards regulating graft survival. IL-22 may make a protective contribution at IRI in allograft liver transplantation by promoting regeneration and proliferation, anti-apoptosis and repair of injury functions for the graft. Meanwhile, persistent over-expression of IL-22 would lead to pathogenic functions for the graft at AR stage, because IL-22 can also induce the inflammatory response, increasing chemokines, recruiting inflammatory factors like Th17, and reducing Treg cells.

In summary, as shown in Figure 7, after allograft liver transplantation, IL-22 is secreted by CD4^+^ T cells mainly, but possibly by other multiple cell sources as well, such as γδ T cells and ILCs, all of which are or would be stimulated by the acute injection process. Through activation of the STAT3 signaling pathway, IL-22 plays a dual role in regulating graft survival. IL-22 may make a protective contribution at IRI stage of allograft liver transplantation by promoting regeneration and proliferation, anti-apoptosis and repair of injury functions for the graft. Whereas persistent over-expression of IL-22 would lead to pathogenic effects on allografted liver tissues at AR stage by up-regulation of chemokines and other inflammatory signals, and recruitment of pathogenic effector cells (e.g. Th17) and decrease of immune suppressive cells (e.g. Treg) to the inflamed tissues.

## MATERIALS AND METHODS

### Animals and liver transplantation

Inbred male BN (RT1^n^) rats and LEW (RT1^1^) rats (SPF grade, 200–240g) [22] were purchased from the Animal Center at Third Military Medical University (Chongqing, China). The rats were housed in a temperature and light controlled environment with free access to tap water and standard rat chow. Orthotopic liver transplantation was performed under ether anesthesia according to Kamada’s method [40] with a few modifications. Recipient rats were randomly divided into 2 groups: the BN-to-LEW isograft group (n=25), and the LEW-to-BN allograft group (n=45). After operation, food and water were available *ad libitum* and no further treatment was given. Sacrifice of rats (5 from each group) for analysis was made on the day before operation or on day 1, 3, 5 and 7 postoperatively. In addition, recipient rats of the allograft group received pretreatment with neutralizing IL-22 antibody, IL-2 antibody or control IgG, and were sacrificed on day 1 and 7 after liver transplantation (5 for each subgroup). Peripheral blood and liver graft tissues were obtained for further experimental analysis at the indicated time points. All procedures in this study were approved by the Institutional Animal Care and Use Committee of the Third Military Medical University.

### Liver histology

Liver tissue was fixed in 10% neutral-buffered formalin and embedded in paraffin. The 4-μm sections were stained with hematoxylin and eosin (HE). The histological classification was analyzed blindly using the rejection activity index (RAI) according to Banff’s scheme. [41]

### Biochemical analysis

Plasma levels of alanine aminotransferase (ALT), aspartate aminotransferase (AST) and total bilirubin (TBIL) were determined by an automated chemical analyzer (Olympus, Tokyo, Japan).

### Enzyme-linked immunosorbent assay (ELISA)

Plasma concentrations of IL-22 and IL-17 were determined by ELISA kits (R&D Systems, Minneapolis, MN, USA). All ELISAs were performed according to the manufacturer’s instructions. All samples were measured in duplicate.

### Quantitative polymerase chain reaction (qPCR)

Total liver RNA was isolated by using Trizol reagent (Invitrogen; Carlsbad, CA, USA) according to the standard protocol. RNA was reverse transcribed with oligo-dT primer using Superscript II (Invitrogen). Real-time PCR was performed using the SYBR Green PCR Master Mix and RT-PCR kit (Applied Biosystems, Foster City, CA, USA) according to the manufacturer’s instructions. All samples were normalized to β-actin mRNA. The primers’ sequences for RT-PCR are as follows:

IL-22, forward 5′-TTCCGAGGAGTCAAAG CCA-3′, reverse 5′-CGCAGGGACATAAACAGCA-3′;

IL-22R1, forward 5′-CTACGTGTGCCGAGTG AAG-3′, reverse 5′-GCGTAGGGGTTGAAAGGT-3′;

IL-10R2, forward 5′-GCCAGCTCTAGGAAT GAT-3′, reverse 5′-AATGTTCTTCAAGGTCCAC-3′;

IL-13, forward 5′-ATCGAGGAGCTGAGCAAC AT-3′, reverse 5′-ATCCGAGGCCTTTTGGTTAC-3′;

IL-17, forward 5′-CAATCCCACGAAATCCAGG AT-3′, reverse 5′-GGTGGAGATTCCAAGGTGAG-3′;

Bcl-xL, forward 5′-GCTGCATTGTTCCCGTAG AG-3′, reverse 5′-GTTGGATGGCCACCTATCTG-3′;

Cyclin D1, forward 5′-TCAGGAGCTCCAAAG CAACT-3′, reverse 5′-TTCTTCATCGGGAGCTGGT-3′;

FOXP3, forward 5′-ACCGTATCTCCTGAGTTC-3′, reverse 5′-GTCCAGCTTGACCACAGTT-3′;

CXCL10, forward 5′-TCAGCACCATGAACCCA AG-3′, reverse 5′-CTATGGCCCTCATTCTCACTG-3′; CCL20, forward 5′-AGACAGATGGCCGATGAAG-3′, reverse 5′-TCTTGACTCTTAGGCTGAGGA-3′;

CXCR3, forward 5′-TTGCCCTCCCAGATTTC ATC-3′, reverse 5′-TGGCATTGAGGCGCTGAT-3′; CCR4, forward 5′-TATTGCAAGGCAAAGACTAT-3′, reverse 5′-GATTTACTCCATCAGCCAGT-3′;

CCL22, forward 5′-TGCCGTATTACGTCCGT TA-3′, reverse 5′-TCTGAGGTCCAGTAGAAGTGT-3′;

β-actin, forward 5′-AGGGAAATCGTGCGTGAC-3′ and reverse 5′-GGAAGGAAGGCTGGAAGAG-3′.

### Immunohistochemistry (IHC) and immunofluorescent (IF) staining

Fresh liver tissues were embedded in OCT compound (Tissue Tek, Tokyo, Japan) and stored at −80°C until use. Six-micrometer cryostat sections were prepared for staining. Briefly, the sections were fixed in acetone (−20°C, 5 min), air-dried, and blocked with 1% BSA. The specimens were then incubated with goat polyclonal IL-22 antibody or rabbit polyclonal IL-17 antibody (Santa Cruz Biotechnology, Dallas, TX, USA) overnight at 4°C. The sections were washed and incubated for 1h with a polymeric, peroxidase-labeled secondary antibody (Dako, Copenhagen, Denmark). Reactivity was detected with a DAB Elite Kit (Dako), and brown coloration of tissues indicated positive staining. And five different high power fields (400 ×) of liver portal areas were used for counting positive cells by two independent observers. For IF double-staining, the sections were incubated overnight with goat polyclonal IL-22 antibody and mouse monoclonal CD4 antibody (Santa Cruz Biotechnology), followed by fluorescence-conjugated secondary antibody (CY5 or FITC labeled anti-goat or anti-mouse IgG; Santa Cruz Biotechnology). Nuclei were counter-stained with DAPI.

### Flow cytometry

Rat liver lymphocyte were processed with the Optiprep Nycoprep Lymphoprep Kit (Axis-Shield, Oslo, Norway), and stained with the indicated antibodies for assessment by flow cytometry. For assessment of the cell surface molecules, the anti-CD3 FITC and anti-CD4 APC antibodies (eBioscience, San Diego, CA, USA) were used. The Fixation/Permeabilization Kit (eBioscience) was used to immune-stain the intracellular molecules with anti-IL-22 PE, anti-IL-17 PE and anti-FOXP3 PE (eBioscience). For IL-22 andIL-17 staining, the cells were pretreated using the following procedure: cells were first cultured in DMEM and then stimulated for 6 h with 50ng/mL PMA (Sigma, St. Louis, MO, USA) and 500ng/mL ionomycin (Sigma) in the presence of 1μL/mL GolgiStop (BD Biosciences, San Diego, CA, USA). FACS analyses were carried out with a FACSAria cell sorter (BD Biosciences) and FlowJo analysis software (Tree Star, Ashland, OR, USA).

### Western blotting

Protein was extracted from liver tissue using RIPA buffer on ice. The extracted protein sample was mixed in loading buffer, boiled for 5 min, and resolved by SDS-PAGE. After electrophoresis, proteins were transferred onto PVDF membranes (Millipore, Billerica, MA, USA) in TRIS/glycine buffer. Primary antibody blotting was performed using goat polyclonal IL-22 antibody, mouse monoclonal IL-22R1/p-STAT3/STAT3/β-actin antibody (Santa Cruz Biotechnology). Secondary antibody included anti-goat HRP or anti-mouse HRP-linked IgG. Detection was performed with the Super Signal West Pico Substrate System (Thermo Fisher Scientific, Rockford, MD, USA).

### Neutralizing antibody administration

Animals were treated with neutralizing antibody by intravenous injection twice, at 12 and 24 h prior to sacrifice on days 1 and 7 postoperatively. Each recipient was administered 100μg rabbit anti-mouse IL-22 or IL-2 polyclonal antibody or normal rabbit IgG (Sungene Biothech, Tianjin, China) each time.

### Statistical analysis

All statistical analyses were performed using GraphPad Prism software (GraphPad Software, San Diego, CA, USA). Differences between groups were analyzed using Student’s paired *t*-test or analysis of variance. *P* value of <0.05 was considered significant.

## SUPPLEMENTARY MATERIALS FIGURES AND TABLES



## Abbreviations

ALT: alanine aminotransferase
AST: aspartate aminotransferase
ELISA: enzyme-linked immunosorbent assay
FOXP3: Forkhead box P3
GVHD: graft versus host disease
HCC: hepatocellular carcinomas
HE: hematoxylin and eosin
ILCs: innate lymphoid cells
IFNγ: interferon-gamma
IL: interleukin
IHC: immunohistochemistry
IF: immunofluorescent
JAK: Janus kinase
NF-κB: nuclear factor-κB
NK: natural killer cells
NKT: natural killer T cells
qPCR: quantitative polymerase chain reaction
RORγt: retinoid-related orphan nuclear receptor γt
Treg cells: regulatory T cells
RAI: rejection activity index
STAT3: the signal transducer and activator of transcription factor 3
TBIL: total bilirubin
