# Supplementary material for: Dual roles of IL-22 at ischemia-reperfusion injury and acute rejection stages of rat allograft liver transplantation
Source: Oncotarget. 2017 Dec 15;8(70):115384–97. doi: 10.18632/oncotarget.23266 (PMC5777779; doi:10.18632/oncotarget.23266)
Supplement: Supplementary file 1 [file oncotarget-08-115384-s001.pdf]

Dual roles of IL-22 at ischemia-reperfusion injury and acute rejection stages of rat allograft liver transplantation

SUPPLEMENTARY MATERIALS

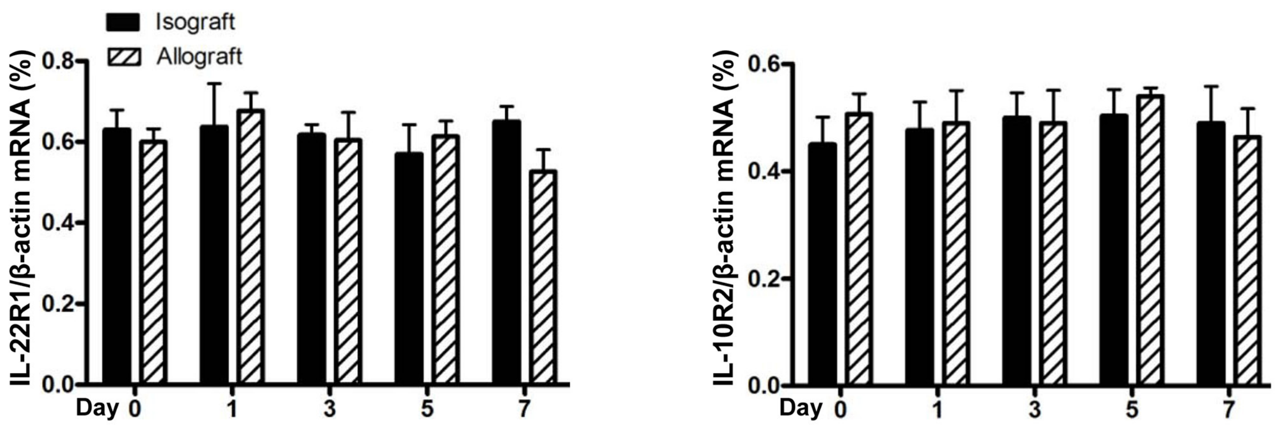

Supplementary Figure 1: Expression levels of IL-22 receptor in allogeneic and isogenic liver transplant tissues. IL-22R1 and IL-10R2 mRNA levels in liver tissues of recipients (n=5) in the isograft group and the allograft group before or at the 1, 3, 5 and 7 days after liver transplantation.

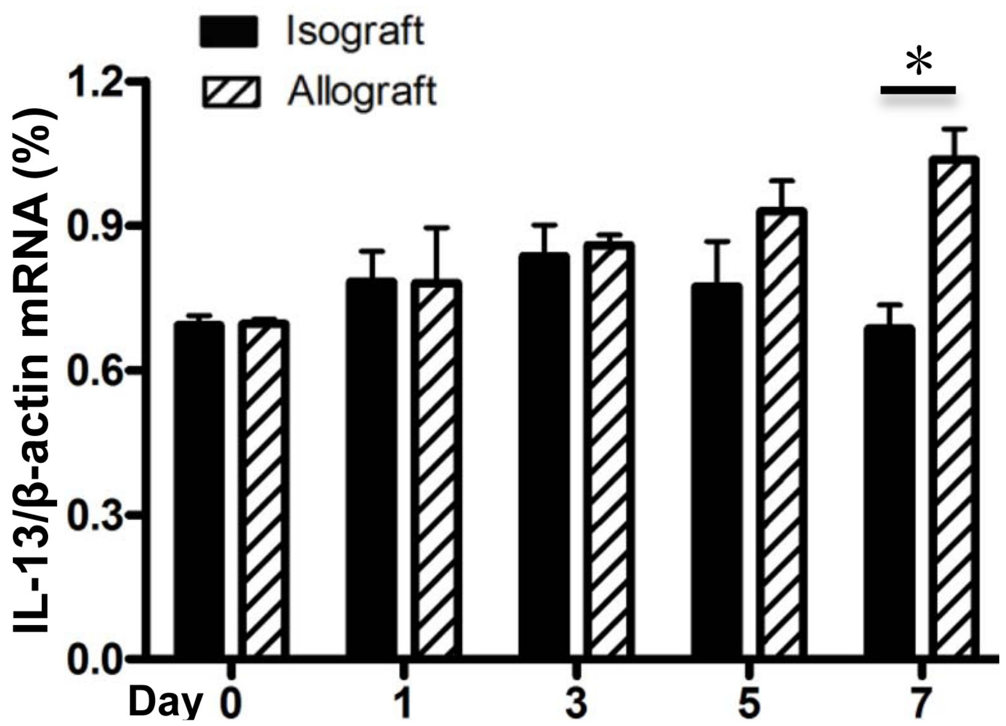

Supplementary Figure 2: IL-13 expression in allogeneic and isogenic liver transplant tissues. IL-13 mRNA levels in liver tissues of recipients (n=5) in the isograft or allograft group at the indicated time points were determined by quantitative RT-PCR. *, $p<0.05$.
